# How to Deal with Another Man's Patients

**Published:** 1890-10

**Authors:** 


ARTICLE VIII.

### HOW TO DEAL WITH ANOTHER MAN'S PATIENTS.

Few professional men have been so blessed as to have passed through their career without being forced into unpleasantness through misunderstandings arising out of their relations with other men's patients. In many ways this occurs: A. goes away and asks B. to look after casual callers during his (A.'s) absence on a holiday or through illness. Patients, possibly new ones to A., but recommended to his house by other patients, come to B., like him and wish to remain with him, and allege they do not know A. and do not desire to leave B. having once gone to him. Take another case: C. has a misfortune and either gives a patient more pain than he, the patient, thinks necessary and so betakes himself to another dentist D., again recommended by friends. It also happens that some measure is suggested by one dentist which is not to the taste of a patient and so the latter hies him off to get another dentist's opinion, often enough not mentioning names until the opinion is expressed, and if this is unfavorable to the previously proposed operation the patient may elect to remain with No. 2 dentist and forsake No. 1.

Children will often enough lead their parents to change their dentist, because Mr. So-and-So is so "rough to poor little Willie." A bad smash at one house followed by a successful extraction elsewhere may lead to alienation of patient and dentist, even if the smash was absolutely unavoidable and the lucky extraction the fruit of fortuitous inflammation plus manual dexterity, leaving it an open question which factor played the larger part in the happy result. Under any and all of these circumstances how is the dentist who is appealed to in the second place to act, and what must his demeanor be towards his predecessor whose patients the people were before they came to him? Certainly, no item of professional ethics bristles with more difficulties or presents larger scope for the exercise of tact and judicial honesty. It is too often lost sight of that doctors and dentists alike have no vested interests in their patients, the practice of giving money for the goodwill of a practice gave rise to the idea, but undoubtedly, patients have an absolute right to change their dentist as often as they please, and at the present day they are more than ever disposed to act upon their rights and seek aid in their dental troubles where they consider they can most satisfactorily obtain it. Patient grabbing is quite another thing to accepting a patient who deliberately selects between A. and B. and prefers B., although, in this case, unless there is a definite cause of complaint against A., it is certainly B.'s duty to point out that the patient being recommended to A. is *ipso facto* his, and to send the person on to A. upon his return to the charge of his practice. Where patient-grabbing comes in is when a dentist asked casually to look in a mouth, volunteers a sneer or faint praise of work already placed in the mouth. In every case it is accepted by professional men as being quite outside the bounds of etiquette to criticise unfavorably the work of another duly qualified practitioner, and the offence is aggravated when the criticism is offered without solicitation. Probably, no better advice upon this most vexed question in professional ethics can be given than that implied in the dictum, "do to others as you would that they should do to you." However, there are very many side issues which need ventilation and being well threshed out, which can very well be done through the columns of our "Correspondence," and we shall be glad if our readers will give their brethren the benefit of their experience upon the matter. Many complain that assistants as "grabbers," and not a few assistants, turn the tables by reporting "sharp practice" by former principals. Even allowing for the Briton's privilege to grumble, we think the question may well be looked at from all points of view.—*Editorial, London Dental Record.*

